# Fragmented Perception: Slower Space-Based but Faster Object-Based Attention in Recent-Onset Psychosis with and without Schizophrenia

**DOI:** 10.1371/journal.pone.0059983

**Published:** 2013-03-25

**Authors:** Henderikus G. O. M. Smid, Richard Bruggeman, Sander Martens

**Affiliations:** 1 University Center of Psychiatry, University Medical Center Groningen, Groningen, The Netherlands; 2 Neuroimaging Center, University of Groningen, Groningen, The Netherlands; 3 Department of Neuroscience, University Medical Center Groningen, Groningen, The Netherlands; Chiba University Center for Forensic Mental Health, Japan

## Abstract

**Background:**

Schizophrenia is associated with impairments of the perception of objects, but how this affects higher cognitive functions, whether this impairment is already present after recent onset of psychosis, and whether it is specific for schizophrenia related psychosis, is not clear. We therefore tested the hypothesis that because schizophrenia is associated with impaired object perception, schizophrenia patients should differ in shifting attention between objects compared to healthy controls. To test this hypothesis, a task was used that allowed us to separately observe space-based and object-based covert orienting of attention. To examine whether impairment of object-based visual attention is related to higher order cognitive functions, standard neuropsychological tests were also administered.

**Method:**

Patients with recent onset psychosis and normal controls performed the attention task, in which space- and object-based attention shifts were induced by cue-target sequences that required reorienting of attention within an object, or reorienting attention between objects.

**Results:**

Patients with and without schizophrenia showed slower than normal spatial attention shifts, but the object-based component of attention shifts in patients was smaller than normal. Schizophrenia was specifically associated with slowed right-to-left attention shifts. Reorienting speed was significantly correlated with verbal memory scores in controls, and with visual attention scores in patients, but not with speed-of-processing scores in either group.

**Conclusions:**

deficits of object-perception and spatial attention shifting are not only associated with schizophrenia, but are common to all psychosis patients. Schizophrenia patients only differed by having abnormally slow right-to-left visual field reorienting. Deficits of object-perception and spatial attention shifting are already present after recent onset of psychosis. Studies investigating visual spatial attention should take into account the separable effects of space-based and object-based shifting of attention. Impaired reorienting in patients was related to impaired visual attention, but not to deficits of processing speed and verbal memory.

## Introduction

Intact perception of objects is fundamental to all human activity but is compromised by schizophrenia [1]. Schizophrenia is a debilitating brain disorder, usually accompanied by recurrent psychotic episodes consisting of hallucinations, delusions, and impaired insight. To date, cognitive research of schizophrenia has emphasized deficits of attention, memory, and executive function, but disrupted perceptual processing also has been amply demonstrated with possibly serious consequences for higher-order cognitive functions [2]. Normal perception of an object evolves from sensory elements that are bound together by pre-attentive mechanisms according to Gestalt principles, together referred to as the perceptual organization process [3]. Clinical and experimental evidence demonstrates that in schizophrenia this process is impaired, resulting in loosened figure-to-ground organization, deteriorated perception of an object as an integrated whole, and possibly in a source for deriving delusional meaning from a scene [1], [4], [5]. For example, reporting the separate elements of visually presented stimuli normally deteriorates when the Gestalt-based organization of the stimulus increases. Schizophrenia patients, however, are hardly affected by the level of organization [6]. Other studies point to deficits of object recognition [7] and perceptual closure [8] in these patients.

Normal operation of many higher cognitive functions depend on intact object perception, for example the ability to identify and recognize objects, and to shift attention between objects in the visual field. We therefore predicted that because schizophrenia is associated with impaired object perception, schizophrenia patients should show a deficit of shifting attention between objects. To test this prediction, we applied a task [9], that makes it possible to separately observe space-based and object-based shifting of attention. Impairment of object perception has been observed especially in relation to psychotic symptoms. Psychosis, however, not only accompanies schizophrenia, but also other psychiatric and somatic conditions. Since it is not clear whether impaired object perception is specific for schizophrenia, and whether object perception is already impaired after a recent onset of psychosis, recent onset psychosis patients (ROP) with and without schizophrenia, and healthy controls (HC) were included in the present experiment. Given the evidence implying associations between impaired object perception, psychotic symptoms, and higher cognitive functions, understanding this deficit is important for understanding psychosis.

Orienting of visual attention is a well-studied and conceptualized form of selective attention, involving the ability to disengage, move, and focus attention in visual space [10], [11]. In the classical test [10], participants are cued somewhere in the visual field for the probable location of an upcoming target stimulus to which they have to respond. On no-shift trials the target is presented in the same location as the cue, and on shift trials it appears in another location, requiring reorienting of attention. Typically, Reaction Times (RTs) are slower on shift than on no-shift trials, providing a measure of the time required to disengage attention from the cued location and to move it to the target location [12]. In its original form, however, this task confounds space-based with object-based attention, because it typically uses outline-boxes or circles as indicators where in the visual field cues and targets appear. Thus, on shift trials the participants not only have to reorient attention across a certain distance in space, but also from an attended object to an unattended one, which usually takes more time [3], [9], [13], [14].

**Figure 1:**
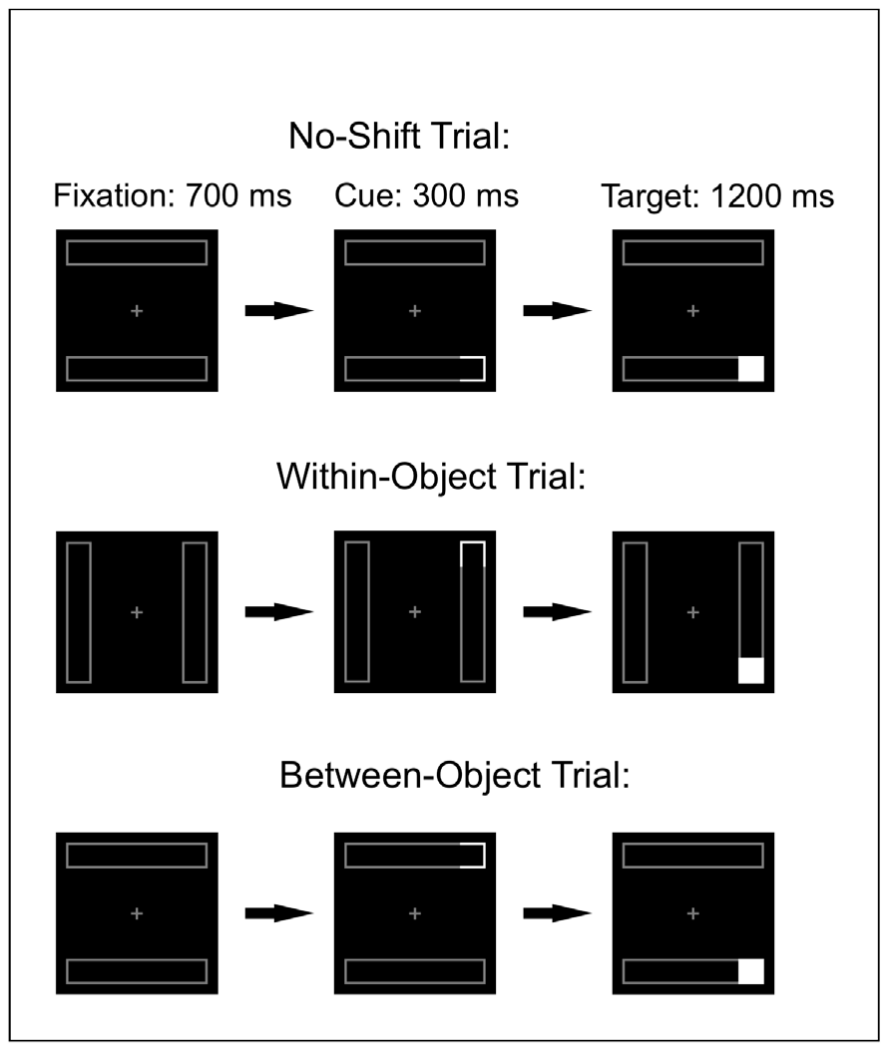
Stimulus sequence examples in no-shift (upper), within-object (middle) and between-object (lower) trials. Participants responded manually to detection of the solid white square.

To avoid this confounding, we applied a task [9] that previously has been successfully used to observe object-based attention impairments in parietal lesion patients, split-brain patients [15] and dyslexia [16]. Reorienting attention from cue to target on some trials occurred within an object (a rectangle), and on other trials the cue was presented on one object and the target on another object, with the same spatial distance as in the within-object trials (See Fig. 1 and Method for details). Typically, the Reaction Times (RTs) on within-object trials are delayed relative to trials on which cue and target are presented at the same location. This reflects the time it takes for attention to disengage from the cued location and to move to the target location within the cued object, that is a space-based shift of visual attention. In between-object trials, RTs are usually more delayed than in within-object trials, demonstrating that it takes more time to disengage and move attention from one object to another object than to disengage and move attention across the same distance within an object. This additional cost shows that movement of visual attention is sensitive to object representations in the visual field and can be delayed by them, producing an object-based RT component [9]. Thus, if objects are less well represented in schizophrenia patients, the difference in RT between within-object and between-object trials, i.e., the object-based component of RT, would be expected to be *smaller* than in controls (see Discussion for situations in which RT may be *delayed* as a consequence of impaired object perception).

To gain more insight in neuropsychological impairments associated with schizophrenia, we examined how space- and object-based attention shifting are related to higher-order neuropsychological function domains [17]. Meta-analyses show that schizophrenia is most strongly associated with impairments of verbal memory, speed of information processing, and attention [17], [18], [19]. In healthy controls, auditory speech shadowing interferes with visual reorienting, suggesting that visual attention shares limited resources with attention to auditory verbal information [20]. This, and other evidence [21] suggest that verbal memory deficits in psychosis may be related to a deficit of visual attention. Further, the nature of speeded visual scanning tasks in the speed of processing domain (e.g., Stroop Word- and Color-Naming), suggests that space- and object-based attention deficits would also be correlated with this domain, as well as with selective attention (e.g., Stroop Color-Word Naming).

## Methods

### Participants

Fifty-three in- and out-patients (37 males) were recruited from the University Center of Psychiatry at the University Medical Center Groningen and twenty-seven HC (16 males) through advertisements. Inclusion criteria were an age between 18 and 40 years and the presence of an episode of psychosis in the preceding 24 months, Exclusion criteria were a history of neurological or psychiatric disorders of the participant or a first relative, vision problems after correction, and drug dependence. Starting at the time of admission to the University Center, the patients underwent an 8-week diagnostic protocol as part of standard-care procedures. Some of these patients were referred on the basis of acute psychosis while others were referred for re-assessment of their status in longitudinal care, ensured by the regular contact between clinicians and patients, and required by the mental health care system in the Netherlands for this group of patients. In this 8-week protocol, the data from clinical-diagnostic interviews, observations, heteroamnestic interviews and clinical records of the referring clinics and general practitioners were applied by SCAN trained senior psychiatrists to test in consensus the DSM-IV criteria for the presence of a psychosis in the preceding two years and for the disorder underlying that psychosis. Time of onset of first symptoms and start of anti-psychotic treatment were recorded. The Positive and Negative Symptoms Scale (PANSS), obtained within in a week distance of the week of testing, was used to assess the severity of current psychotic symptoms. Premorbid education level was scored on the basis of the highest level finished at the time of recruitment, with scores ranging from 1 (primary school) to 7 (university).

Of the 53 ROP, 27 had a DSM-IV 295.xx diagnosis of schizophrenia (paranoid n = 20; schizophreniform n = 5; schizoaffective n = 1; undifferentiated n = 1). The other 26 had other than 295.xx diagnoses of psychosis (Psychotic Disorder NOS or Brief Psychotic Disorder n = 12; Bipolar Disorder n = 12; Delusional Disorder n = 1; Major Depression n = 1). The majority of the patients used antipsychotics (risperidone n = 20, olanzapine n = 16, quetiapine n = 4, clozapine n = 1, perphenazine n = 1) with an averaged mean chlorpromazine (CPZ) equivalent [22] of 244.97 mg/d (SD 102.06). Twelve patients used additional medication (benzodiazapines n = 4, anti-depressives n = 6, anti-cholinergica n = 1, anti-epileptics n = 1) and eleven patients were drug-free during time of testing. Demographic and clinical data are presented in Table 1.

**Table 1:** Group Means (SD) of Demographic and clinical data, Neuropsychological test scores, and Median Target Reaction Times.

	Scores				P-values t-tests			
	HC n = 27	ROP n = 53	NROP n = 26	SROP n = 27	HC-ROP	HC-NROP	HC-SROP	NROP-SROP
**Age**	**26.4 (6.4)**	**25.1 (6.9)**	**27.2 (8.4)**	**23.1 (4.1)**	**Ns**	**Ns**	**0.02654**	**0.02936**
Education	4.2 (1.3)	3.9 (1.6)	4.0 (1.8)	3.7 (1.4)	Ns	Ns	Ns	Ns
IQ	98,3 (15,9)	94.8 (11,8)	97.4 (11.8)	92.3 (11.6)	Ns	Ns	Ns	Ns
PANSS Pos	N/A	11.7 (4.5)	12.1 (5.3)	11.4 (3.7)	N/A	N/A	N/A	Ns
**PANSS Neg**	**N/A**	**13.0 (5.1)**	**11.2 (4.4)**	**14.8 (5.3)**	**N/A**	**N/A**	**N/A**	**0.00971**
PANSS Gen	N/A	27.6 (6.9)	27.2 (8.0)	28.1 (5.9)	N/A	N/A	N/A	Ns
Dis-P2	N/A	1.7 (1.0)	1.9 (1.3)	1.5 (0.7)	N/A	N/A	N/A	Ns
Dis-CogSyn	N/A	1.7 (0.6)	1.8 (0.7)	1.6 (0.6)	N/A	N/A	N/A	Ns
Dis-5Fact	N/A	1.7 (0.5)	1.7 (0.6)	1.6 (0.5)	N/A	N/A	N/A	Ns
**Duration of Illness (weeks)**	**N/A**	**35.0 (24.8)**	**27.4 (19.8)**	**42.2 (27.3)**	**N/A**	**N/A**	**N/A**	**0.0496**
Duration of AP Treatment (weeks)	N/A	9.6 (12.3)	6.4 (5.8)	12.4 (15.6)	N/A	N/A	N/A	Ns
CPZ eq dose/d	N/A	245.0 (102.1)	273.3 (121.3)	223.8 (81.3)	N/A	N/A	N/A	Ns
N using AP:	N/A	42	18	24				
**Str Speed**	**46.2 (7.2)**	**53.5 (9.6)**	**53.4 (9.9)**	**53.6 (9.5)**	**0.00084**	**0.00397**	**0.00211**	**Ns**
Str Interf	32.5 (12.4)	37.9 (17.5)	39.4 (16.2)	36.6 (18.8)	Ns	Ns	Ns	Ns
**Trl Speed**	**27.7 (6.2)**	**38.5 (12.8)**	**35.7 (10.9)**	**41.3 (14.1)**	**0.00000**	**0.00176**	**0.00005**	**Ns**
Trl Interf	13.1 (11.2)	14.5 (17.1)	14.1 (14.9)	14.0 (19.2)	Ns	Ns	Ns	Ns
**CVLT**	**52.9 (11.6)**	**44.9 (8.0)**	**47.9 (7.0)**	**42.1 (7.9)**	**0.00271**	**Ns**	**0.00020**	**0.00691**
**CPT d'**	**4.18 (0.4)**	**3.5 (0.6)**	**3.6 (0.5)**	**3.4 (0.8)**	**0.00000**	**0.00001**	**0.00006**	**Ns**
**FingerTp**	**51.7 (4.7)**	**48.7 (7.5)**	**49.0 (7.3)**	**48.4 (7.8)**	**0.03180**	**Ns**	**Ns**	**Ns**
No-Shift RT	307.6 (41.2)	333.0 (68.3)	328.8 (67.8)	337.1 (69.9)	Ns	Ns	Ns	Ns
**W-Obj RT**	**358.3 (52.8)**	**401.0 (76.2)**	**396.1 (77.4)**	**405.6 (76.2)**	**0.01106**	**0.04218**	**0.01060**	**Ns**
**B-Obj RT**	**380.8 (43.4)**	**413.9 (75.5)**	**408.2 (75.2)**	**419.3 (76.9)**	**0.01527**	**Ns**	**0.02892**	**Ns**
**BORT - WORT**	**22.5 (20.3)**	**12.9 (14.1)**	**12.1 (13.3)**	**13.7 (15.0)**	**0.01557**	**0.03236**	**Ns**	**Ns**

*Note:* all tests corrected for heteroscedacity; HC, healthy controls; ROP, Recent Onset Psychosis patients; NROP, ROP no schizophrenia; SROP, ROP with schizophrenia; Ns, p>.05; IQ, Intelligence Quotient; PANSS Pos, PANSS Positive scale; PANSS Neg, PANSS Negative scale; PANSS Gen, PANSS General scale; Dis-P2, score on PANSS P2 item Conceptual Disorganization; Dis-CogSyn, Disorganization score on Cognitive Syndrome factor; Dis-5Fact, Disorganization factor score; AP, Antipsychotic; CPZ eq dose/d, Chlorpromazine equivalent dose per day; Str, Stroop test; Trl, Trailmaking test; CVLT, California Verbal Learning Test; CPT d', Continuous Performance Test d-prime; FingerTp, Fingertapping test; RT, Reaction Time; W-Obj, within-object; B-Obj, between-object; BORT – WORT, between-object RT minus within-object RT;

Exclusion criteria were checked with a questionnaire. The study was approved by the Ethics Committee of the University Medical Center of Groningen. All subjects were assessed for the capacity to consent and found to be capable to do so by Dr. R. Bruggeman (MD, PhD) and Dr. H. Knegtering (MD, PhD). HC, ROP with schizophrenia (SROP), and ROP with other diagnoses (NROP) did not differ in education (all t<1.4, all p>.16) and intelligence (all t<1.58, all p>.12). SROP were a little younger than NROP (t51 = 2.27, p = .029) and than HC (t52 = 2.28, p = .026). SROP and NROP did not differ in CPZ equivalent dose/d (t51<1), and in the duration of anti-psychotic treatment (t51 = −1.88, p = .07). SROP had a longer duration of illness than NROP (t51 = −2.02, p = .05) and had higher scores on the negative symptom scale of the PANSS than NROP (t51 = −2.69, p = .01). These differences are consistent, showing that a diagnosis of schizophrenia as required by the DSM-IV implies a much longer duration of illness before treatment and more severe negative symptoms than other psychotic disorders. Note in Table 1, that the PANSS scores indicate that for most of the patients the severity of psychosis was low or in remission at the time of testing. Cognitive disorganization as a symptom or symptom factor may be related to cognitive deficits in neuropsychological tasks [23]. There are, however, several ways to assess disorganization based on PANNS items. We therefore used three parameters. The first was simply the score on the Conceptual Disorganization item of the positive symptom scale of the PANSS (P2). The second was the Cognitive Syndrome factor [23] and involves the mean of the scores on the PANSS items P2, N5 and G11. The third concerned the Disorganization factor obtained in a 5-factor analysis of the PANSS items ([24], mean of items P2, N5, N7, G5, G9, G10, G11, G12, G13, G15). As Table 1 shows, SROP and NROP did not differ on any of these three disorganization parameters.

### Materials and procedure

Each trial in the task started with a fixation display consisting of two rectangles with a fixation cross. The two rectangles occupied an imaginary square of 11.4×11.4 degree of visual angle, and were either oriented horizontally or vertically with the fixation aid in the middle. The possible cue-target locations, that is, the four ends of the two rectangles, occupied the same locations in the horizontal and vertical arrangements, 6.8° from fixation. The rectangles remained on the screen for the entire trial. Brightness of the rectangle-outline was dimmed to 50% of the black-to-white greyscale (see Figure 1).

The trial started with presentation of the fixation display for 700 ms. Next, a cue was presented for 300 ms, consisting of a white brightening of the outline of one of the four ends of the two rectangles (100% white on the black-to-white scale; 1.7×1.7°). Finally, the target was presented for 1200 ms, consisting of a white-filled square (1.7×1.7°) in one of the four ends of the two rectangles. The task of the participants was to fixate the fixation cross, to suppress eye-movements, and to press a button located at the body's midline central to the screen with the index finger of the preferred hand as soon as they detected a white-filled square anywhere in the visual field. If the experimenter observed eye-movements, she repeated the eye-movement instruction. There were three types of trials. On no-shift trials, the target appeared at the same location as the cue, but on shift trials, the target appeared either at the other end of the cued rectangle (within-object trials), or at the equidistant end of the other, uncued rectangle (between-object trials).

Participants first performed two short practice blocks of one minute each. Next, they completed five experimental blocks, each consisting of 104 trials. Of these, 53.9% were no-shift trials, 15.4% were within-object trials (target presented in the same rectangle but in its other end), and 15.4% were between-object trials, (target presented in the other rectangle on the end closest to the cue). To discourage anticipations, 15.4% of the trials were nogo trials (a cue not followed by a target). Location of the cue, order of trials and vertical or horizontal rectangles, were randomized. Half of the shift trials required a horizontal shift, half a vertical shift. A total of 80 shift trials of each type was available for analysis.

The neuropsychological battery was administered by experienced test-psychologists according to standard procedures. It was part of the usual care offered to the patients in order to assess their cognitive functioning and the tests are commonly used in research on psychosis. It consisted of the California Verbal Learning Test (Verbal Learning and Memory; CVLT, Dutch translation; measure: total number of items recalled in five trials), the Continuous Performance Test (Visual attention; 3–7 version; measure: d-prime, i.e. Hit-rate corrected for False Alarm rate), The Stroop test (Selective attention; Word-Naming, Color-Naming and Color-Word naming; measures: performance time in sec; interference computed as: Color-Word Naming−[Word-Naming+Color-Naming]/2), the Trailmaking test (Divided visual attention; Digit, Alphabet, and Alternate Digit-Alphabet Trailing; measures: performance time in sec; interference computed as: Alternate Digit-Alphabet−[Digit+Alphabet]/2), Finger Tapping (Motor speed; measure: mean number of single-handed taps per 10 sec over five repetitions with each hand), and a subset of the WAIS-III intelligence test (Information, Arithmetic, Symbol Substitution, and Block Design, [25]). The average of the Word- and Color-Naming scores and of the Digit- and Alphabet-Trailing scores were used as composite measures of processing speed.

### Analyses

For each participant and each location and condition in the within-subject design the median RT was computed [9], [15]. RTs less than 150 ms (i.e., anticipations; ROP: 2.09%, HC: 2.17%), missed targets and false alarm RTs in nogo trials (ROP: 1.85%, HC: 1.57%) were not analyzed. Next, the RT data were analyzed in two different ways. In the first, an omnibus MANOVA was done to demonstrate the effects of diagnosis and overall cuing on the raw median RTs. This overall MANOVA was followed by two pre-planned MANOVAs to test (1) the effects of diagnosis and no-shift versus within-object shift (i.e., the spatial component of shift RT), and (2) the effects of diagnosis and within-object shift versus between-object shift (i.e., the object-based component of shift RT).

Visual field factors, however, have specific effects on target detection time [26], [27]. For example, RTs to targets in the upper visual field are longer than in the lower visual field. Moreover, schizophrenia patients may show a different effect of visual field on target detection than controls [28]. A second analysis was therefore done to cancel-out the effect of visual location on the difference between no-shift and shift RTs. To that end, the median of the no-shift RTs at a particular location was subtracted from the median of the shift RTs at that very same location. This was done separately for each participant, for each of the four target locations, for cued and uncued objects, and for horizontal and vertical attention shifts. In this way, we obtained the RT cost of having to move attention to a location relative to when that location is already occupied by attention, that is, an estimate of the time needed for disengagement and movement of attention. These RT costs were entered in a MANOVA to demonstrate the effect of diagnosis on purely spatial attention shifts and that on the sum of spatial and object-to-object attention shifts.

The significance level of the statistical tests was p = .05. Neuropsychological test-scores were subjected to between-groups t-tests (two-tailed) and non-parametric correlations with attention shifting measures and anti-psychotic medication. Partial correlations were used to control these for age and intelligence.

## Results

### The effects of cuing and group

The omnibus five-way MANOVA on the raw median RTs tested the effects of Cuing (no-shift, within-object, between-object), Rectangle Orientation (horizontal, vertical), Horizontal Field (left, right), and Vertical Field (lower, upper) as within-subject factors and Group (HC, ROP) as between-subjects factor. Here, we focus on the effects of Cuing, Group and their interaction. The effects of visual field factors are presented separately further below.

**Figure 2:**
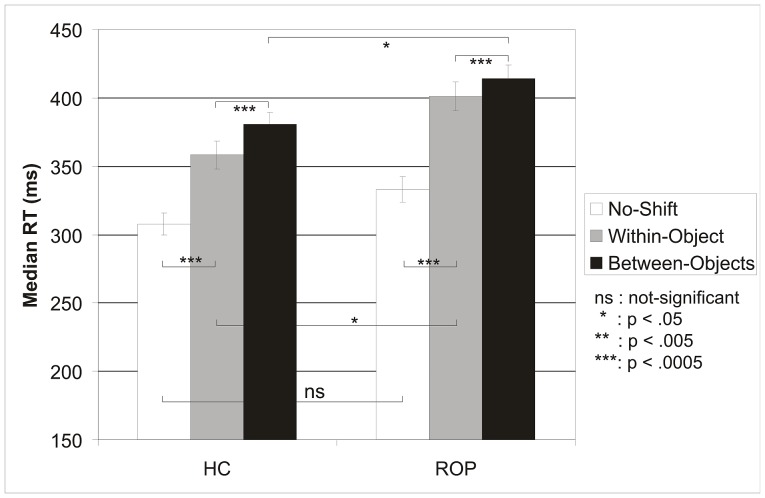
Median RTs (SEM) to no-shift, within-object and between-object trials for healthy controls and all patients.

Figure 2 illustrates the effects of cuing on the median RTs in the two groups. A significant main effect of Group showed that ROP had longer target detection RTs (383 ms) than HC (349 ms; F1,78 = 4.91, p<.03, E = .059). The main effect of Cuing was highly significant (F2,77 = 365.96, p<.0005, E = .91) and a significant Cuing by Group interaction indicated that the cuing effects were different for ROP and HC (F2,77 = 5.036, p<.009, E = .116). As Figure 2 shows, the difference in RTs between no-shift and within-object trials was larger for ROP (333 ms vs. 401 ms) than for HC (308 ms vs. 358 ms). The first pre-planned follow-up MANOVA contrasting no-shift and within-object RTs supported this observation by revealing a highly significant Cuing by Group interaction (F1,78 = 8.167, p<.005, E = .095). These results indicate that ROP needed more time for a purely spatial attention shift than HC. As Figure 2 also shows, the difference in RTs between within-object and between-object trials was smaller for ROP (401 ms vs 414 ms, i.e., 13 ms) than for HC (358 ms vs. 381 ms, i.e., 23 ms). This was supported by the second pre-planned follow-up MANOVA that contrasted within-object and between-object RTs, by a significant Cuing by Group interaction (F1,78 = 6.116, p<.016, E = .073). This demonstrates that having to shift attention between objects across the same distance had less impact on the RT of ROP than on that of HC. We repeated this analysis to observe whether NROP and SROP differed with respect to cuing. In this analysis type of cuing (no-shift, within-object, between-object) was also highly significant (F2,50 = 286.169 p<.0005, E = .920), but did not differ between NROP and SROP (Cuing by Group: p>.8). A separate analysis of the no-shift RTs revealed that these did not differ significantly between HC, NROP and SROP.

**Figure 3:**
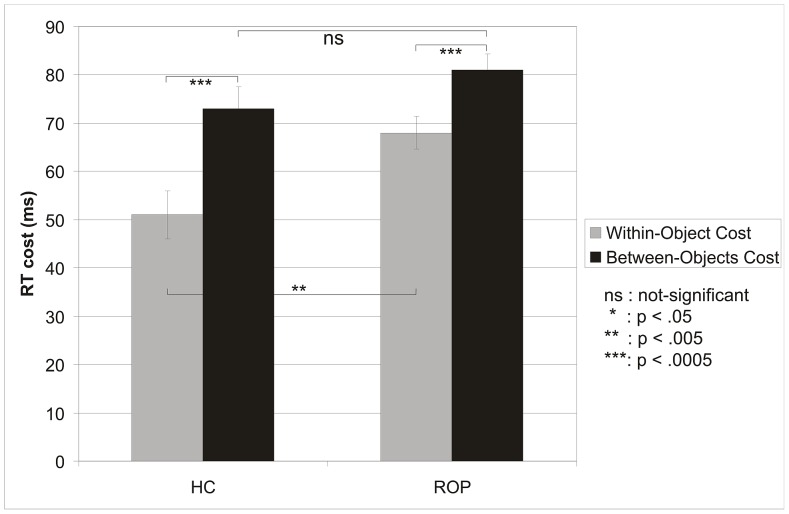
Mean RT costs (SEM) for spatial shifts and object shifts of attention in controls and all patients.

Figure 3 illustrates the RT costs of having to shift attention from the cued location to the target location in the within-object and between-object trials (i.e., no-shift RT subtracted). Note, that within-object RT cost represents the time cost of a purely spatial attention shift, while between-object RT cost represents the summed time costs of having to shift attention across space *and* between objects. These RT costs were analyzed in a MANOVA with Rectangle (cued, uncued), attention Shift Direction (horizontal, vertical), Horizontal Field (left, right) and Vertical Field (lower, upper) as within-subject factors and Group (HC, ROP) as between-subjects factor. There was a main effect of Rectangle cuing. Reorienting within a cued object cost 59 ms and reorienting between objects cost significantly more (77 ms; F1,78 = 83.21, p<.0005, E = .516). A significant main effect of Group (see Figure 3) signified that ROP had overall larger shifting costs (74 ms) than HC (62 ms, F1,78 = 5.07, p<.027, E = .061). A significant Cuing by Group interaction showed that patients had only 13 ms larger shifting costs to uncued rectangles than to cued rectangles, whereas for controls this difference was larger (23 ms; F1,78 = 6.12, p<.016, E = .073; see Figure 3). Follow-up analyses showed that for the cued rectangles ROP had significantly larger shifting costs (68 ms) than HC (51 ms; F1,78 = 8.167, p<.005, E = .095), whereas the cost for the uncued rectangles was not significantly different between the groups (81 ms and 73 ms; p>.18). For each group the main effect of Rectangle cuing was highly significant (HC: F1,26 = 33.252, p<.0005, E = .561; NROP: F1,25 = 21.512, p<.0005, E = .463; SROP: F1,26 = 22.366, p<.0005, E = .462). No other effect involving Rectangle cuing reached significance. When we repeated this analysis to observe differences between NROP and SROP in RT costs, we found a significant main effect of Rectangle cuing (F1,51 = 43.596, p<.0005, E = .461; cued: 68 ms, uncued: 81 ms), but no Rectangle by Group interaction, nor a main Group effect (Fs<1).

### The effects of visual field

In the initial MANOVA on the raw median RTs the only significant visual field effect on the Cuing by Group interaction concerned a Cuing by Rectangle Orientation by Vertical Field by Group interaction (F2,77 = 3.803, p<.027, E = .09). This interaction signifies that patients detected targets always a little faster in the lower than in the upper visual field, while for controls this vertical asymmetry was modulated by rectangle orientation. After controlling for visual location in the RT cost analyses, no visual field factor interacted with the Cuing by Group interactions.

Visual field factors influenced RT costs in ROP and HC. There was a main effect of Vertical Field (F1,78 = 9.05, p<.004, E = .104) and a Shift Direction×Vertical Field×Group interaction (F1,78 = 5.27, p<.024, E = .063), indicating that patients reoriented 13 ms slower upward than downward, whereas controls hardly differed in up- and downward reorienting. Visual field effects on costs differed in NROP and SROP. A Shift Direction×Horizontal Field×Group interaction (F1,51 = 4.355, p<.042, E = .079) indicated that vertical shifts hardly differed in time between left and right hemifields and between groups (largest difference was 5 ms), while for horizontal shifts SROP patients had 16 ms larger shifting times for right-to-left shifts than for left-to-right shifts, whereas NROP had only 6 ms larger right-to-left shifting times. Only the SROP had a significant Shift Direction by Horizontal Field effect (F1,26 = 13.018, p<.001, E = .334, NROP: F<1).

### Neuropsychological test performance

Table 1 presents the neuropsychological test-results. Patients significantly underperformed on the Stroop and Trailmaking speed scores, CVLT recall, CPT d-prime, and Finger tapping. SROP only differed from NROP in having lower CVLT recall scores. In HC, no-shift RT was negatively correlated with CPT d-prime (−.42, p = .029), while shifting costs in RT were negatively correlated only with CVLT performance (spatial: −.48, p = .011, object: −.51, p = .006). In patients, no-shift RT was negatively correlated with CPT d-prime (−.55, p = .003) and Fingertapping (NROP: −.65, p = .0005, SROP: −.58, p = .002). Shifting costs for patients were correlated only with measures of higher order visual attention (for NROP CPT d-prime with spatial: −.53, p = .005, d-prime with object: −.62, p = .001; for SROP d-prime with object: −.50, p = .007; Trailmaking interference for NROP object: .40, p = .042, for SROP spatial: .43, p = .026 and object: .59, p = .001; Stroop interference for SROP object: .42, p = .029). Note, that the size of these correlations was medium (>.30) to large (>.50), most of them large [29], explaining up to 42% of variance between no-shift RT and fingertapping in NROP.

### Correlations with clinical data

Correlations between chlorpromazine equivalent dose/day and neuropsychological testscores were only significant for Stroop Color-Word Naming (.399, p<.003), Stroop interference (.401, p<.003), Trailmaking Digit (.277, p<.045) and Fingertapping (−.377, p<.005). None of the RT measures was significantly correlated with medication dose. Partial correlations controlling for age and intelligence did not change this pattern, except for the correlation with Trailmaking Digit becoming insignificant.

Non-parametric correlations were computed between illness duration, treatment duration, PANSS scale scores, and the three disorganization scores as explained in the Method section on the one hand, and the RT scores from the experimental task on the other hand. That is, with no-shift, within-object and between object RTs, and with the shifting costs within and between objects. None of these correlations was significant. The largest Rho present was between the PANSS P2 item score (Conceptual Disorganization) and within-object shift cost (−.198, p = .15), while all other ps were larger.

## Discussion

The main goal of the present experiment was to test the hypothesis that if schizophrenia is associated with impaired object perception, schizophrenia patients should have an impairment of object-based attention. If objects are less well represented in the visual field, they should have less impact on visual attention mechanisms, resulting in faster than normal shifting speed. Applying a visual covert attention task that makes it possible to separately observe space-based and object-based reorienting of attention, we predicted that the object-based component of RT would be *smaller* in patients than in HC. The results replicated the original findings with this task [9] and confirmed this novel prediction. The analysis of the raw median RTs showed that the difference in RTs between within-object and between-object trials was significantly smaller for ROP (401 ms vs. 414 ms, i.e. 13 ms) than for HC (358 ms vs. 381 ms, i.e., 23 ms), demonstrating that the object-based component of RT was indeed smaller in patients (Fig. 2). The opposite was found for the space-based component of RT. The difference in RTs between no-shift and within-object trials was significantly larger for ROP (333 ms vs. 401 ms, i.e., 68 ms) than for HC (308 ms vs. 358 ms, i.e., 50 ms), demonstrating that the space-based component of RT was larger in patients.

The analysis of the RT costs of invalid cuing, required to control for visual location and between-group effects on no-shift trials, substantiated these findings. The shifting cost for reorienting within cued rectangles (within-object minus no-shift RT, i.e., space-based reorienting), was significantly larger for ROP (68 ms) than for HC (50 ms), but the cost for reorienting to uncued rectangles (between-object minus no-shift RT, i.e., the *sum* of space-based and object based reorienting) was not significantly different between ROP (81 ms) and HC (74 ms). Together, these findings indicate that both space-based and object-based attention are impaired in ROP, but in opposite directions. Space-based reorienting was 17 ms slower, while the object-based component of reorienting was 10 ms smaller for ROP (13 ms) than for HC (23 ms). As a result, the total cost of space- and object-based reorienting (i.e., between-object RT minus no-shift RT) did not significantly differ between ROP and HC (<8 ms). None of these effects was significantly influenced by visual field factors, nor by overall weakening of the ROP attention system, evidenced by the finding that no-shift RTs did not significantly differ between ROP and HC. These findings show that the visual attention system of ROP is less influenced by the presence of objects in the visual field than that of healthy controls, and support the hypothesis that psychosis disorders are associated with an impairment of the perceptual organization underlying object perception [1]. Importantly, they show that perceptual deficits can have pronounced effects on higher-order cognitive functions like the orienting of attention.

The between-object component of RT was smaller for ROP than for HC. This indicates that for ROP the objects interfered less with the movement of attention than for HC. This effect seems limited, however, to tasks in which the objects are irrelevant for response decision, as in the present task. Participants had to respond as fast as possible to the presentation of any white filled square, irrespective of its relationship to the rectangles and cue. In tasks in which objects are to be attended for response decision, the opposite might be found. In this case, impaired object perception may increase the time for object identification, and thereby increase RT relative to HC. Thus, effects of impaired object perception in ROP on task performance may depend on the extent to which the objects presented in the task require attention for deciding on responses. In the present task, reorienting was exogenously-directed, involuntarily and reflexive, but in tasks in which it must endogenously be directed (e.g., by a central arrow, or searching a display with objects), impaired object perception may result in slowed task performance.

This idea may have consequences for our interpretation of the abnormally increased RTs on within-object shift trials of patients relative to their no-shift RTs. Object-based attention is conceived as the result of spreading attention to all locations in the cued object and at the cost of less attention to the uncued object [30]. Normally, this would speed-up attention shifts within the cued object compared to an uncued object. If patients suffer from a lack of attention spreading in the cued object, this speed-up may not occur, resulting in abnormally delayed within-object attention shifts. In short, both the slower within-object performance and the faster object-based component of performance by the patients relative to the controls may be viewed as the result of deficient object-based attention mechanisms. We found, however, that patients and controls did not differ significantly in the trials in which also part of an object was cued, but no further shift within the object was required, i.e., in the no-shift trials. This argues against this alternative interpretation, because if patients basically have less spread of attention in an object, then significantly slower RTs in no-shift trials would be expected compared to the HC. This is a complex matter, and whether this alternative interpretation is correct or not, the present findings would still support the hypothesis that psychosis is associated with impaired object representation, having serious consequences for higher-order cognitive processes like visual attention.

One way to further investigate this issue may be to apply some form of neutral cue or baseline condition. In exogenous cuing tasks (contrary to endogenous ones), however, there seems to be no convincing neutral cue possible because every conceivable cue would lead to within or between object shifts or a difference in cue intensity [12]. One suggestion for further research could be to apply a base-line condition in which at the same locations as in the present ones cues and targets are presented, but without any objects present in the visual field.

The second goal of this experiment was to investigate whether abnormalities of object perception are already present after a recent psychotic episode, and whether they are specific to patients with schizophrenia. For SROP and NROP significant abnormal slowing of space-based and abnormal speeding of object-based reorienting were observed, but no differences between the groups. This suggests that both space-based reorienting and object perception are abnormal already after a recent episode of psychosis, and is not only associated with schizophrenia. The only difference in target detection performance between NROP and SROP consisted of a horizontal field asymmetry in reorienting (from right-to-left slower than from left-to-right) for SROP, but this pattern was not present for NROP. This suggests that moving attention from right-to-left is abnormal in schizophrenia. Since vertical within-hemifield reorienting did not differ between groups nor between hemifields, the abnormal movement from the right field seems to arise only when attention has to cross the vertical meridian, possibly related to abnormal unidirectional transcallosal transfer. Previous evidence on horizontal asymmetry in schizophrenia is conflicting [20], [28], [31], [32]. Both NROP and SROP had an abnormal vertical asymmetry, consisting of slower up- than downward reorienting relative to controls. Thus, a vertical asymmetry seems associated with psychosis in general, but both a vertical and a horizontal asymmetry seem associated with a diagnosis of schizophrenia.

Our findings shed a different light on previous findings with reorienting attention tasks, that, as far as we are aware of, always used objects like squares and circles as cue- and target-containers and therefore always required reorienting between objects [20], [28], [31], [32], [33], [34], [35], [36], [37], [38], [39]. As our results show, these studies confounded space-based and object-based reorienting costs. We found that ROP were slower than HC in space-based reorienting, but had a shorter object-based reorienting component, with the net result that ROP and HC did not significantly differ in the total cost of space- and object-based reorienting. Previous studies on spatial attention may therefore have systematically underestimated the slowing of space-based reorienting in patients, because it was masked by the faster object-based component. This may explain the many inconsistent findings of these studies, for example that patients needed more time than controls for reorienting attention [20], [28], that they needed less time [39], or did not differ in reorienting speed [36].

Neuropsychological performance of patients showed abnormal processing speed, verbal memory, transient attention, and motor speed, consistent with the extant literature [17]. Of these, only motor speed was correlated with medication dose. Patients with schizophrenia were special, in that they had worse verbal memory scores than patients without schizophrenia and controls. Visual attention measures were correlated with neuropsychological measures, but in different ways in patients and controls. In controls, larger shifting costs were only associated with worse verbal memory scores, consistent with previous word-shadowing findings [20], and suggesting that visual attention and attention to verbal information share common resources. In psychosis patients, however, larger shifting costs were only associated with worse transient (CPT), selective (Stroop) and divided (Trailmaking) visual attention. This suggests that the verbal memory deficit in patients is not related to attention, consistent with several studies that have pointed at slowed processing speed as an important component of verbal memory deficits in schizophrenia [40]. It seems therefore that the memory and processing speed impairments involve related deficits that are separate, maybe independent, from visual attention impairments.

The present findings allow for four conclusions. First, deficits of object perception and attention shifting are not only associated with schizophrenia, but are common to all patients suffering from psychosis. SROP only differed from NROP by having abnormally slow right-to-left visual field reorienting. Secondly, deficits of object perception and visual attention shifting are already present after a recent episode of psychosis. Thirdly, investigation of visual spatial attention should take into account the separable effects of space-based and object-based shifting of attention. Finally, impaired reorienting in patients is related to impaired visual attention, but not to deficits of processing speed and verbal memory.
